# ELAVL1 regulates glycolysis in nasopharyngeal carcinoma cells through the HMGB3/β-catenin axis

**DOI:** 10.1186/s10020-024-00941-5

**Published:** 2024-10-10

**Authors:** Yi Cui, Haojie Wen, Jinyong Tang, Jiawen Chen, Juan Zhou, Minghua Hou, Xiaohan Rong, Yuanzhao Lan, Qiong Wu

**Affiliations:** 1 Department of Otorhinolaryngology Head and Neck Surgery, The First People’s Hospital of Chenzhou (Affiliated Chenzhou Hospital, Southern Medical University), Chenzhou, Hunan 423000 P.R. China; 2 Department of Otorhinolaryngology Head and Neck Surgery, The First Affiliated Hospital of Xiangnan University, Chenzhou, Hunan 423000 P.R. China; 3 Department of Nephrology, The First People’s Hospital of Chenzhou (Affiliated Chenzhou Hospital, Southern Medical University), No. 102, luojiajing, beihu District, Chenzhou, Hunan 423000 P.R. China

**Keywords:** ELAVL1, Glycolysis, Nasopharyngeal carcinoma, HMGB3, Β-catenin

## Abstract

**Background:**

The role of ELAVL1 in the progression of various tumors has been demonstrated. Our research aims to investigate how ELAVL1 controls the glycolytic process in nasopharyngeal carcinoma cells through the HMGB3/β-catenin pathway.

**Methods:**

The expression of ELAVL1 was detected in clinical tumor samples and nasopharyngeal carcinoma cell lines. A subcutaneous tumor model was established in nude mice to investigate the role of ELAVL1 in tumor progression. The relationship between HMGB3 and ELAVL1 was validated by RNA pull down and RIP assays. TOPFlash/FOPFlash reporter assay was used to detect β-catenin activity. Assay kits were utilized to measure glucose consumption, lactate production, and G6PD activity in nasopharyngeal carcinoma cells. Western blot was conducted to detect the expression of glycolysis-related proteins. The glycolytic capacity was analyzed through extracellular acidification rate (ECAR).

**Results:**

In both clinical samples and nasopharyngeal carcinoma cell lines, the expression levels of ELAVL1 mRNA and protein were found to be upregulated. Knockdown of ELAVL1 significantly inhibited the in vivo proliferation of nasopharyngeal carcinoma and suppressed the glycolytic capacity of nasopharyngeal carcinoma cells. ELAVL1 interacts with HMGB3, leading to an increase in the stability of HMGB3 mRNA. Overexpression of HMGB3 elevated the reduced β-catenin activity caused by sh-ELAVL1 and reversed the inhibitory effect of sh-ELAVL1 on cellular glycolytic capacity. Treatment with β-catenin inhibitor (FH535) effectively suppressed the promotion of glycolytic capacity induced by HMGB3 overexpression.

**Conclusions:**

ELAVL1 promotes glycolysis in nasopharyngeal carcinoma cells by interacting with HMGB3 to stabilize HMGB3 mRNA, thereby activating β-catenin pathway. Therefore, targeting the ELAVL1-HMGB3-β-catenin axis has the potential to be a novel approach for treating nasopharyngeal carcinoma.

**Supplementary Information:**

The online version contains supplementary material available at 10.1186/s10020-024-00941-5.

## Highlights

ELAVL1 promotes glycolysis in nasopharyngeal carcinoma cells.

ELAVL1 interacts with HMGB3, thereby activating β-catenin pathway.

HMGB3 is involved in ELAVL1-mediated glycolysis in nasopharyngeal carcinoma cells.

HMGB3 promotes glycolysis by activating β-catenin pathway in nasopharyngeal carcinoma cells.

**Supplementary Information:**

The online version contains supplementary material available at 10.1186/s10020-024-00941-5.

## Introduction

Nasopharyngeal carcinoma is a malignant tumor originating from the epithelial cells lining the nasopharynx. It has a high incidence rate in East Asia, Southeast Asia, and North Africa(Sung et al. 2021). Over the past three decades, treatment options for nasopharyngeal carcinoma have witnessed continuous advancements. The progress in radiotherapy technology and the widespread use of chemotherapy regimens have significantly enhanced the prognosis of patients with localized nasopharyngeal carcinoma(Wong et al. 2021). Following systemic treatment, early-stage nasopharyngeal carcinoma patients can achieve a 5-year overall survival rate and failure-free survival rate of more than 90%(Pan et al. 2016). However, despite these improvements, around 15-30% of patients still encounter challenges such as distant metastasis and recurrence(Li et al. 2019; Wang et al. 2020), which currently represent the primary causes of treatment failure in nasopharyngeal carcinoma(Sun et al. 2019). Further exploration of nasopharyngeal carcinoma’s pathogenesis is required for identifying novel therapeutic targets and providing valuable insights into its better management.

A hundred years ago, Otto Warburg accidentally discovered that cancer cells exhibited increased glucose uptake and produced large amounts of lactate compared to normal cells, even in the presence of oxygen(Liberti and Locasale 2016). This phenomenon referred to as the Warburg effect, or aerobic glycolysis describes the preference of cancer cells to undergo glycolysis to produce energy and biosynthetic intermediates, providing a growth advantage to cancer cells. Aerobic glycolysis is considered a fundamental characteristic of tumor cell metabolism and an important mode of tumor metabolic reprogramming(Vaupel et al. 2019). Nowadays, numerous regulatory factors and biomarkers associated with glycolytic enzymes have been discovered(Xu et al. 2022). For example, SIX1 is overexpressed in various human cancers, where it can transcriptionally regulate the expression and function of glycolysis-related genes, increasing the activity of key glycolytic enzymes like glucose transporter 1 (GLUT1) and hexokinase 2 (HK2), thereby promoting glycolysis and tumor growth(Li et al. 2018). Similarly, alpha-enolase (ENO1) serves as a crucial biomarker in tumor glycolysis, with significantly increased expression in non-small cell lung cancer, and activates FAK/PI3K/AKT pathway, promoting cell glycolysis, proliferation, migration, and invasion(Fu et al. 2015). Targeting these highly expressed molecules in tumor cells holds promise for achieving metabolism-targeted therapy for cancer while minimizing the impact on normal cell glucose metabolism.

ELAVL1, also referred to as HuR, is a kind of RNA binding protein. It plays a regulatory role in essential cellular responses such as proliferation, stress, and apoptosis, which are crucial for normal growth and development(Majumder et al. 2022; Siomi and Dreyfuss 1997). However, dysregulation of ELAVL1 has been implicated in various diseases, including cancer(Srikantan and Gorospe 2012). Compared to normal tissues, the level of ELAVL1 protein is elevated in various types of cancer, including nasopharyngeal carcinoma(Tian et al. 2023), breast cancer(Heinonen et al. 2011), prostate cancer(Mitsunari et al. 2016), and pancreatic cancer(Richards et al. 2010). Furthermore, increased cytoplasmic levels of ELAVL1 have been correlated with advanced tumor stage and unfavorable outcome(Richards et al. 2010; Bolognani et al. 2012; Melling et al. 2016). Therefore, ELAVL1 is considered a promising target for cancer treatment, and numerous studies have investigated the therapeutic effects of inhibiting ELAVL1(Wu and Xu 2022). However, the role of ELAVL1 in glycolysis in nasopharyngeal carcinoma remains largely unknown.

As a chromatin protein, high mobility group box 3 (HMGB3) can interact with chromatin structures, modify chromatin structure and regulate transcription processes(Wen et al. 2021). Recent studies have provided evidence for the strong association between HMGB3 and tumor occurrence, growth and metastasis(Zhang et al. 2017). Notably, in nasopharyngeal carcinoma cells, HMGB3 expression is significantly upregulated. Knockdown of HMGB3 has been shown to inhibit proliferation, angiogenesis, and metastasis of nasopharyngeal carcinoma(Liu et al. 2020; Zhang et al. 2021). In the study on non-small cell lung cancer cells, Shi et al. found that silencing HMGB3 can suppress hypoxia-induced glycolysis(Shi et al. 2019). Other studies have demonstrated that HMGB1 and HMGB2, members of the same family as HMGB3, can target glycolytic genes, thereby enhancing glycolysis to promote progression in breast and pancreatic cancers(Cai et al. 2017; Chen et al. 2021; Fu et al. 2018). However, there is limited research investigating the regulation of glycolysis by HMGB3 in cells of nasopharyngeal carcinoma. The Wnt/β-catenin pathway is a well-established signaling pathway that plays a significant role in tumorigenesis and the progression of cancer. Recent studies have primarily focused on elucidating the role of the Wnt/β-catenin pathway in tumor cell metabolic reprogramming(El-Sahli et al. 2019). Studies have demonstrated that suppressing the Wnt/β-catenin pathway in cells of colon cancer led to a marked reduction in lactate production and glucose consumption, indicating the involvement of β-catenin in aerobic glycolysis of tumor cells(Pate et al. 2014).

In our previous study, we predicted a potential binding relationship between ELAVL1 and HMGB3 using the ENCORI database, suggesting that ELAVL1 may target and regulate HMGB3 expression. Moreover, it has been demonstrated that HMGB3 can promote tumor proliferation and migration by activating the Wnt/β-catenin pathway(Gong et al. 2022; Xie et al. 2019; Zhuang et al. 2020). These discoveries lead us to propose a hypothesis that ELAVL1 interacts with HMGB3 to enhance its stability, thereby promoting aerobic glycolysis in nasopharyngeal carcinoma cells by further activating the β-catenin pathway.

## Materials and methods

### Dataset analysis

We used the UALCAN (https://ualcan.path.uab.edu/index.html) database to analyze the expression of ELAVL1, HMGB3 in head and neck squamous cell carcinoma, and the Encyclopedia of RNA Interactomes (ENCORI, http://starbase.sysu.edu.cn/) to predict the interaction of ELAVL1 with HMGB3 mRNA.

### Human samples

We collected 30 pairs of nasopharyngeal carcinoma tissues and non-tumor nasopharyngeal epithelial tissues from patients who underwent surgery from June 2021 to March 2023. The fresh tissues were immediately preserved on ice, and RNA was then extracted for quantitative real-time polymerase chain reaction (qRT-PCR) analysis. This research was approved by the Ethics Committee of The First People’s Hospital of Chenzhou (Chenzhou, China), ensuring compliance with ethical standards for human research.

### Cell culture and cell transfection

The experimental cell lines used in this study included human nasal epithelial cells (HNEpC) and human nasopharyngeal carcinoma cell lines (HONE-1, C666-1, CNE-1, and SUNE-1), which were obtained from Cell Bank, Chinese Academy of Sciences (Shanghai, China). These cells were cultured in Dulbecco’s Modified Eagle’s Medium (DMEM; Gibco, Grand Island, NY, USA) supplemented with 10% fetal bovine serum (FBS; Gibco) under standard conditions, including a 5% carbon dioxide atmosphere, 5% air, and a temperature of 37 ℃. To achieve stable knockdown of ELAVL1, cells were transfected with an ELAVL1-shRNA plasmid (RiboBio, Guangzhou, China) using Lipofectamine 3000 (Invitrogen, Carlsbad, CA, USA). Non-targeting shRNA constructs were used as negative controls. Following infection with the lentiviruses, the cells were subjected to puromycin selection (5 mg/mL, Invitrogen) to obtain a homogeneous population of infected cells. The efficiency of transfection and infection was evaluated using qRT-PCR and western blot techniques.

### Xenograft animal model

The xenograft tumor model was established using four-week-old female BALB/c mice. Lentivirus-infected HONE-1 cells were injected subcutaneously into the dorsal region of BALB/c mice at 5 × 10^6^ cells per mouse. Tumor volume was measured once every week, and at the end of five weeks, the mice were euthanized, and the tumors were removed and measured. The tumor volume was calculated using the formula: tumor volume (mm^3^) = 1/2 × length × (width)^2^. The obtained tumor tissues were fixed in polyformaldehyde, embedded in paraffin, and sectioned for further analysis. The Laboratory Animal Ethics Committee of The First People’s Hospital of Chenzhou granted approval for all animal experiments, which were performed in strict accordance with the guidelines approved by the Medical Experimental Animal Management Committee.

### qRT-PCR

Cells and tissues were subjected to total RNA extraction using Trizol reagent (Invitrogen), followed by reverse transcription of the extracted RNA into cDNA using the PrimeScript™ Kit (TaKaRa Bio Inc., Otsu, Tokyo, Japan). The synthesized cDNA was used as a template for qRT-PCR analysis using SYBR Green PCR Master Mix (YEASEN, Shanghai, China). Each group was set up with three technical replicates. The housekeeping gene GAPDH was used as an internal reference gene for normalization. To describe the relative mRNA expression in the experimental group, the 2^−ΔΔCT^ method was employed. The primer sequences were listed as follows: ELAVL1, 5’-TGT TCT CTC GGT TTG GGC GGA T-3’ (forward), 5’-TCT TCT GCC TCC GAC CGT TTG T-3’ (reverse); HMGB3, 5’-CCA AGA AGT GCT CTG AGA GGT G-3’ (forward), 5’-CTT CTT GCC TCC CTT AGC TGG T-3’ (reverse); β-catenin, 5’-CACAAGCAGAGTGCTGAAGGTG-3’ (forward), 5’-GATTCCTGAGAGTCCAAAGACAG-3’ (reverse); GAPDH, 5’-GTC TCC TCT GAC TTC AAC AGC G-3’ (forward), 5’-ACC ACC CTG TTG CTG TAG CCA A-3’ (reverse).

### Western blot

RIPA lysis buffer (Beyotime, Shanghai, China) containing protease inhibitors was used to lyse cells and obtain total proteins. Nuclear protein extraction was performed using a Qproteome Cell Compartment Kit (Qiagen, Duesseldorf, Germany). The protein samples were then added to sodium dodecyl sulfate (SDS) protein buffer, heated at 95℃ for 5 min to ensure complete denaturation of the proteins. The protein samples and markers were loaded into the sample wells of an SDS- polyacrylamide gel electrophoresis (PAGE) gel, followed by electrophoresis, transfer onto a polyvinylidene fluoride (PVDF) membrane (Millipore, Bellerica, MA, USA), and blocking with BSA. Specific primary antibody incubation was performed followed by incubation with secondary antibody sourced from the same species as the primary antibody. Visualization was achieved using an enhanced chemiluminescence (ECL) reagents (ABClonal, Wuhan, China), and the resulting bands on the developed image were subjected to gray scale calculation and statistical analysis using software such as ImageJ. β-actin was used as the control for the normalization of the data. The primary antibodies used in this study include ELAVL1 (1:1000, ab200342, Abcam, Cambridge, MA, USA), HMGB3 (1:5000, ab72544, Abcam), HK2 (1:1000, ab209847, Abcam), GLUT1 (1:200, ab150299, Abcam), lactate dehydrogenase (LDHA; 1:5000, ab52488, Abcam), β-actin (1:10000, ab227387, Abcam), β-catenin (1:5000, ab32572, Abcam), and Lamin B1 (1:2000, ab133741, Abcam).

### Immunohistochemistry

After baking at 60℃ for one hour, tissue sections underwent deparaffinization with xylene and were subsequently rehydrated via a series of alcohol gradients with different concentrations. Antigen retrieval was performed in an EDTA solution at 95℃ for 20 min, and 10% goat serum was used to reduce non-specific background staining. Ki67 primary antibody (1:200, ab16667, Abcam) was applied and incubated overnight at 4℃, followed by incubation with a secondary antibody of the same species for 2 h. DAB (3,3’-diaminobenzidine) chromogen and hematoxylin staining were subsequently performed.

### Measurements of glucose uptake, extracellular lactate secretion, and G6PD activity

The glucose uptake, extracellular lactate secretion, and G6PD activity in individual cell samples were detected using the Glucose Colorimetric Assay Kit (E-BC-K234-M, Elabscience, Wuhan, China), Lactate Colorimetric Assay Kit (E-BC-K044-M, Elabscience), and G6PDH Activity Assay Kit (E-EL-H1816c, Elabscience) according to the manufacturer’s instructions.

### Seahorse analysis

The Seahorse XF Glycolysis Stress Test Kit (Agilent Technologies, Santa Clara, CA, USA) was used to measure Extracellular Acidification Rate (ECAR). The treated cells were seeded in a 96-well plate and cultured for 12 h, 36 h, and 60 h. Glucose (10 mM), oligomycin (1 µM), and 2-Deoxy-D-glucose (2-DG; 50 nM) were sequentially added during the experiment. The Seahorse XF Analyzer (Agilent Technologies) was utilized to dynamically and real-time measure extracellular acidification of the cells. The data was analyzed using Seahorse Wave software.

### RNA pull-down assay

The detection of enriched protein-RNA interactions was accomplished using the Pierce™ Magnetic RNA-Protein Pull-Down Kit (Thermo Scientific™ in the USA). First, the cells were lysed using a lysis buffer to release the cellular proteins. Next, the lysate was incubated with a biotin-labeled HMGB3 RNA probe for a specified period of time to allow the probe to bind to the target protein. The mixture was then subjected to magnetic bead capture to isolate the protein complex bound to the RNA probe. Subsequently, the bound proteins were released from the RNA probe, using conditions such as heating or the addition of a protease. Finally, the quantification of ELAVL1 protein was performed by Western blot analysis to investigate the interaction between HMGB3 mRNA and ELAVL1 protein.

### RNA immunoprecipitation (RIP) assay

First, cells were lysed using RIPA lysis buffer (MCE, CA, USA) and centrifuged to collect the supernatant. Concurrently, agarose beads (Thermo Scientific™, USA) were prepared and coated with ELAVL1 antibody or IgG control antibody. The lysate was then mixed with the beads for immunoprecipitation. Subsequently, magnetic beads were added with equal volume of Trizol to resuspend the mixture for RNA isolation. Finally, quantitative analysis was performed using qRT-PCR. The qRT-PCR results obtained from the ELAVL1 and IgG groups were normalized using the results from Input group. Subsequently, the fold changes of the ELAVL1 group in relation to the IgG group were calculated, referred to as relative HMGB3 enrichment.

### RNA stability assay

Cells from different groups were treated with actinomycin D (5 µg/mL) and collected at 0 h, 3 h, and 6 h time points. Total RNA was extracted from the collected cells and reverse transcribed to synthesize cDNA. The levels of HMGB3 mRNA were then quantitatively detected using qRT-PCR.

### Dual luciferase reporter assay

The activity of β-catenin pathway was detected using dual luciferase reporter with TOPFlash (D2501, Beyotime) and FOPFlash (D2503, Beyotime) plasmids. Each group of cells was distributed into separate wells of a 24-well plate and subjected to transfection with either TOPFlash or FOPFlash for a duration of 24 h. After transfection, cells were incubated with LiCl for 24 h, and the relative luciferase activity was determined using a dual luciferase reporter kit (RG027, Beyotime).

### Statistical analysis

The data were expressed as the mean ± standard deviation (SD) derived from three independent repetitions. One-Way ANOVA followed by the post-comparison of Tukey’s Honestly Significant Difference test (for multiple groups) or Student’s *t*-test (for two groups) was used to analyze the significant difference. The correlation between variables was assessed using Pearson analysis. Statistical analysis was conducted with SPSS 22.0. Statistical significance was determined by a *p* value of less than 0.05.

## Results

### ELAVL1 is highly expressed in nasopharyngeal carcinoma tissues and cell lines

**Fig. 1:**
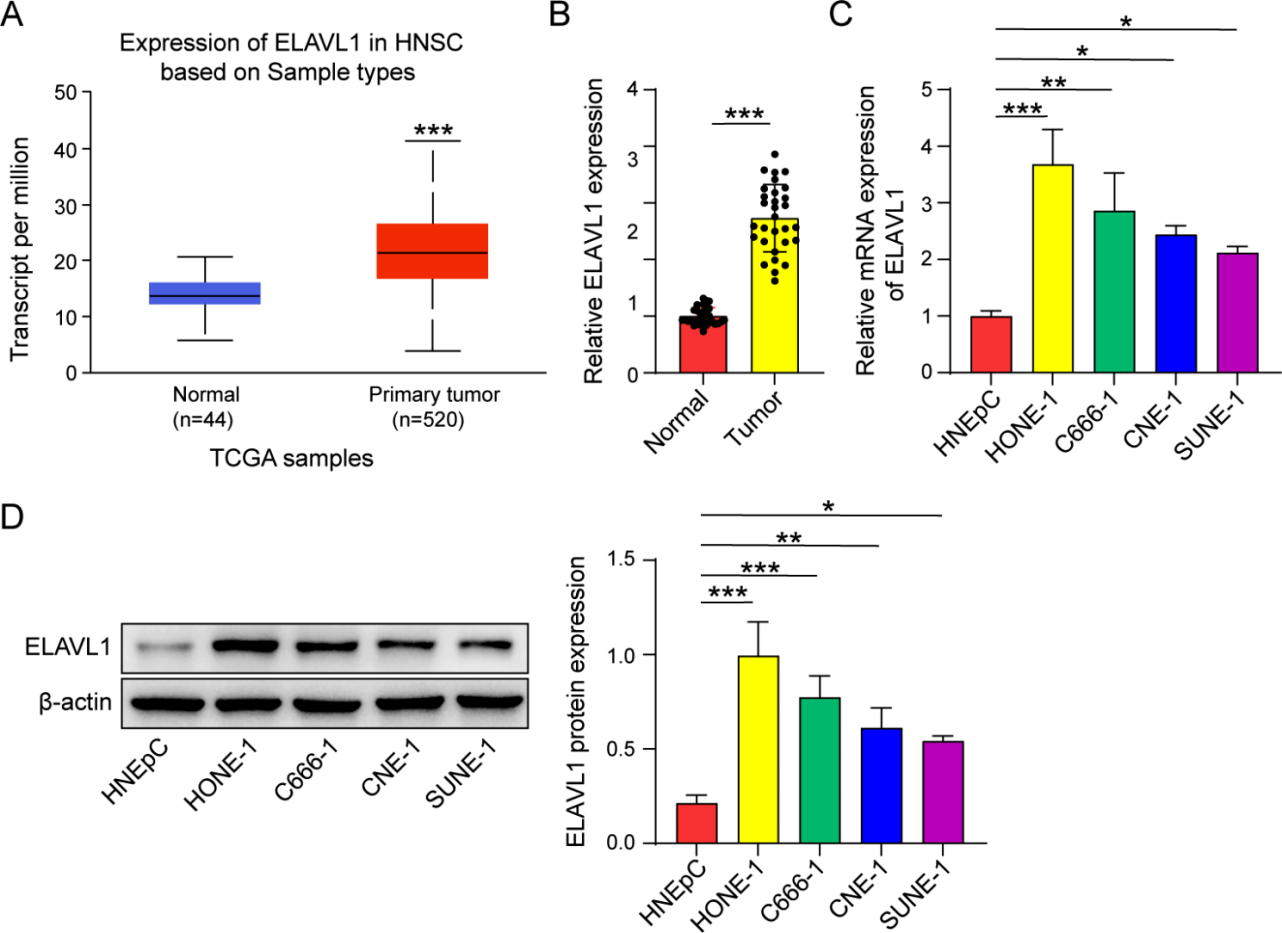
ELAVL1 is highly expressed in nasopharyngeal carcinoma. (**A**) UALCAN analysis of ELAVL1 expression in head and neck squamous cell carcinoma. (**B**) High expression of ELAVL1 in nasopharyngeal carcinoma tissues by qRT-PCR assay. *n* = 30. (**C, D**) ELAVL1 expression levels in nasopharyngeal carcinoma cell lines were measured using qRT-PCR and western blot assays. *n* = 3. *p < 0.05; **p < 0.01; ***p < 0.001

The expression of ELAVL1 was observed to be markedly upregulated in head and neck squamous carcinoma in comparison to non-tumor tissues, as evidenced by data extracted and analyzed from the UALCAN database (Fig. 1A). To further validate this finding, we conducted qRT-PCR analysis to compare the mRNA expression levels of ELAVL1 in nasopharyngeal carcinoma tissues and normal tissues (Fig. 1B). We also performed qRT-PCR and western blot experiments on various in vitro cultured nasopharyngeal carcinoma cell lines, which further substantiated our findings that ELAVL1 expression was considerably higher in nasopharyngeal carcinoma cells than in normal cells, at both the mRNA and protein levels (Fig. 1C, D). Notably, two cell lines demonstrating notably elevated ELAVL1 expression, HONE-1 and C666-1, were specifically chosen for subsequent investigations.

### ELAVL1 promotes glycolysis in nasopharyngeal carcinoma cells

**Fig. 2:**
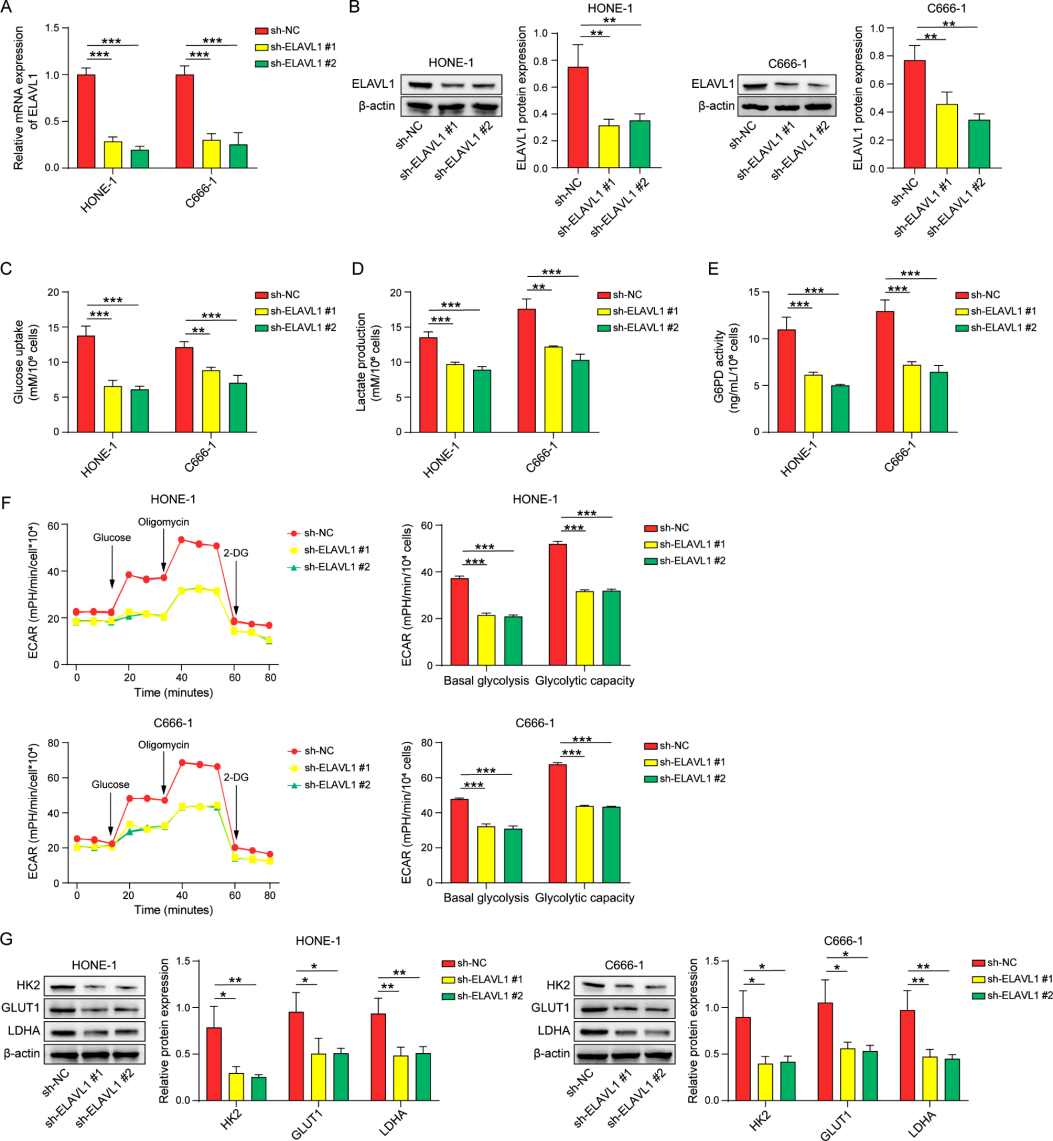
ELAVL1 promotes glycolysis in nasopharyngeal carcinoma cells. (**A**, **B**) The expression of ELAVL1 was assessed using qRT-PCR and western blot assays. (**C-E**) Glucose consumption, lactate production, and G6PD Activity were measured using commercialized kits. (**F**) Basal glycolysis and glycolytic capacity were assessed with extracellular acidification rate (ECAR). (**G**) HK2, GLUT1, and LDHA protein levels were detected by western blot. *n* = 3. *p < 0.05; **p < 0.01; ***p < 0.001

HONE-1 and C666-1 cells were subjected to shRNA-mediated knockdown of ELAVL1, and the impact was assessed at both mRNA and protein levels. The findings demonstrated effective knockdown efficiency (Fig. 2A, B). To further explore the influence of ELAVL1 on glycolysis, several indicators related to glycolysis were analyzed in these cells. The results revealed that ELAVL1 knockdown results in a markedly decrease in glucose consumption (Fig. 2C), inhibition of lactate production (Fig. 2D), and decreased activity of G6PD (Fig. 2E). ECAR assay was performed and showed that ELAVL1 knockdown in HONE-1 and C666-1 cells led to a reduction in both basal glycolysis and glycolytic capacity when compared to the control groups. (Fig. 2F). Additionally, downregulation of ELAVL1 suppressed the expression of glycolytic enzymes, including HK2, GLUT1, and lactate dehydrogenase (LDHA) (Fig. 2G). Collectively, these findings suggest a role for ELAVL1 in promoting glycolysis in nasopharyngeal carcinoma cells.

### ELAVL1 interacts with HMGB3 and increases HMGB3 mRNA stability

**Fig. 3:**
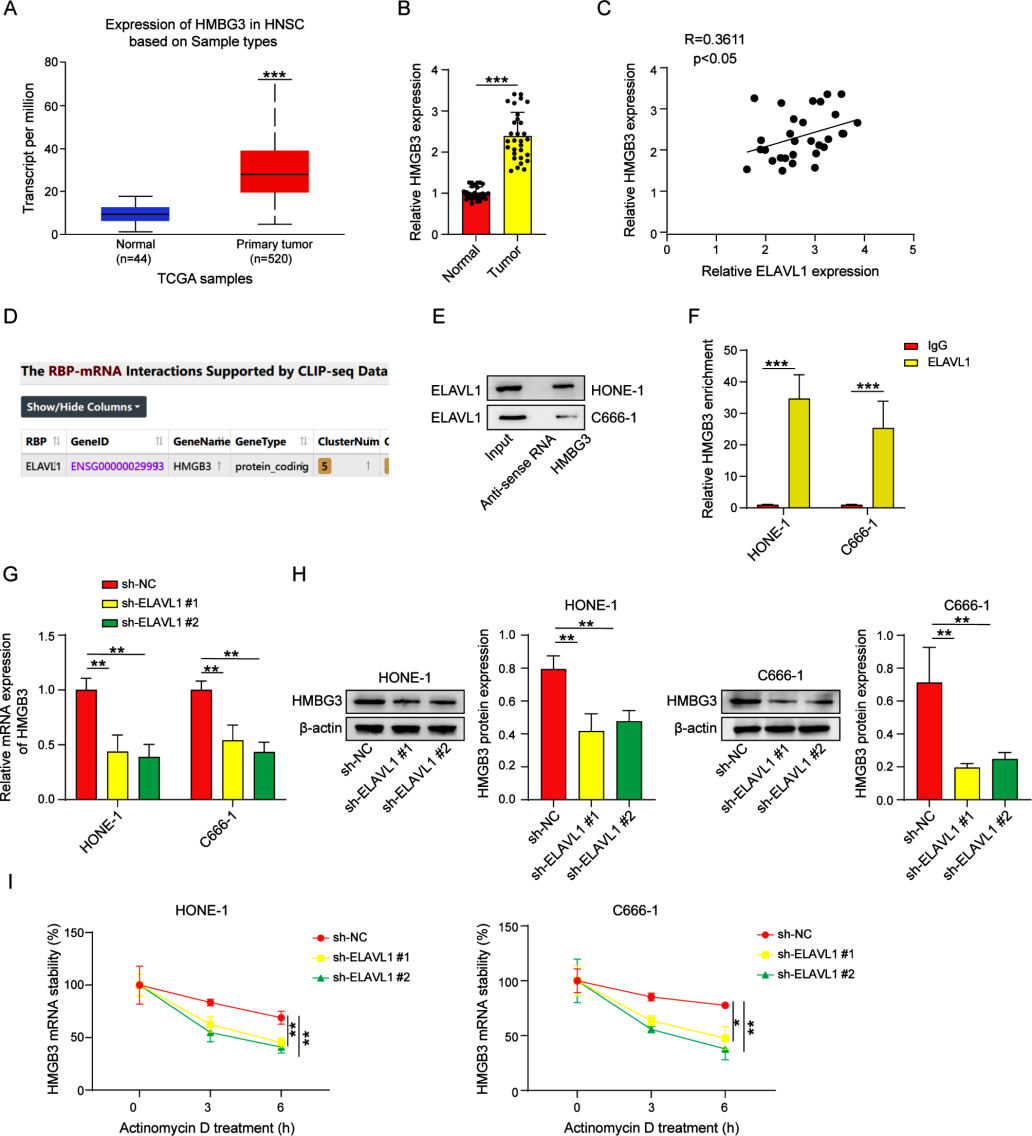
ELAVL1 interacts with HMGB3 and increases HMGB3 mRNA stability. (**A**) UALCAN database analysis of HMGB3 expression in head and neck squamous cell carcinoma. (**B**) The expression of HMGB3 in nasopharyngeal carcinoma tissues was detected by qRT-PCR. (**C**) HMGB3 positively correlates with ELAVL1 expression by Pearson correlation analysis. *n* = 30. (**D**) ENCORI database prediction suggests ELAVL1 binding to HMGB3. (**E**) ELAVL1 binding to HMGB3 mRNA was verified by RNA pull down. (**F**) ELAVL1 and HMGB3 mRNA interaction was validated by RNA immunoprecipitation assay (RIP). (**G**, **H**) The effects of ELAVL1 on HMGB3 mRNA and protein levels were verified by qRT-PCR and western blot. (**I**) HMGB3 mRNA stability was determined by qRT-PCR. *n* = 3. *p < 0.05; **p < 0.01; ***p < 0.001

After analyzing with the UALCAN database, we observed a high expression of HMGB3 in head and neck squamous cell carcinoma (Fig. 3A). By conducting qRT-PCR analysis of 30 pairs of clinical specimens from nasopharyngeal carcinoma and non-tumor patients, the elevated expression of HMGB3 in nasopharyngeal carcinoma was confirmed (Fig. 3B). Furthermore, the strong positive correlation between ELAVL1 and HMGB3 was detected via Pearson correlation analysis (Fig. 3C), suggesting a potential association between HMGB3 and ELAVL1. This finding was further supported by prediction results obtained from the ENCORI database, which indicated a potential binding relationship between ELAVL1 and HMGB3 (Fig. 3D). To validate these findings, pull-down assay was performed in HONE-1 and C666-1 cells, confirming the presence of interaction between ELAVL1 and HMGB3 mRNA (Fig. 3E). Subsequently, RIP assay was conducted, where the ELAVL1 antibody exhibited greater enrichment of HMGB3 mRNA compared to IgG, providing further validation for the interaction between ELAVL1 and HMGB3 (Fig. 3F). Furthermore, knockdown of ELAVL1 significantly decreased the expression of HMGB3 mRNA and protein (Fig. 3G, H). Additionally, we observed that knockdown of ELAVL1 also compromised the stability of HMGB3 mRNA (Fig. 3I).

### ELAVL1 activates the β-catenin pathway through HMGB3

**Fig. 4:**
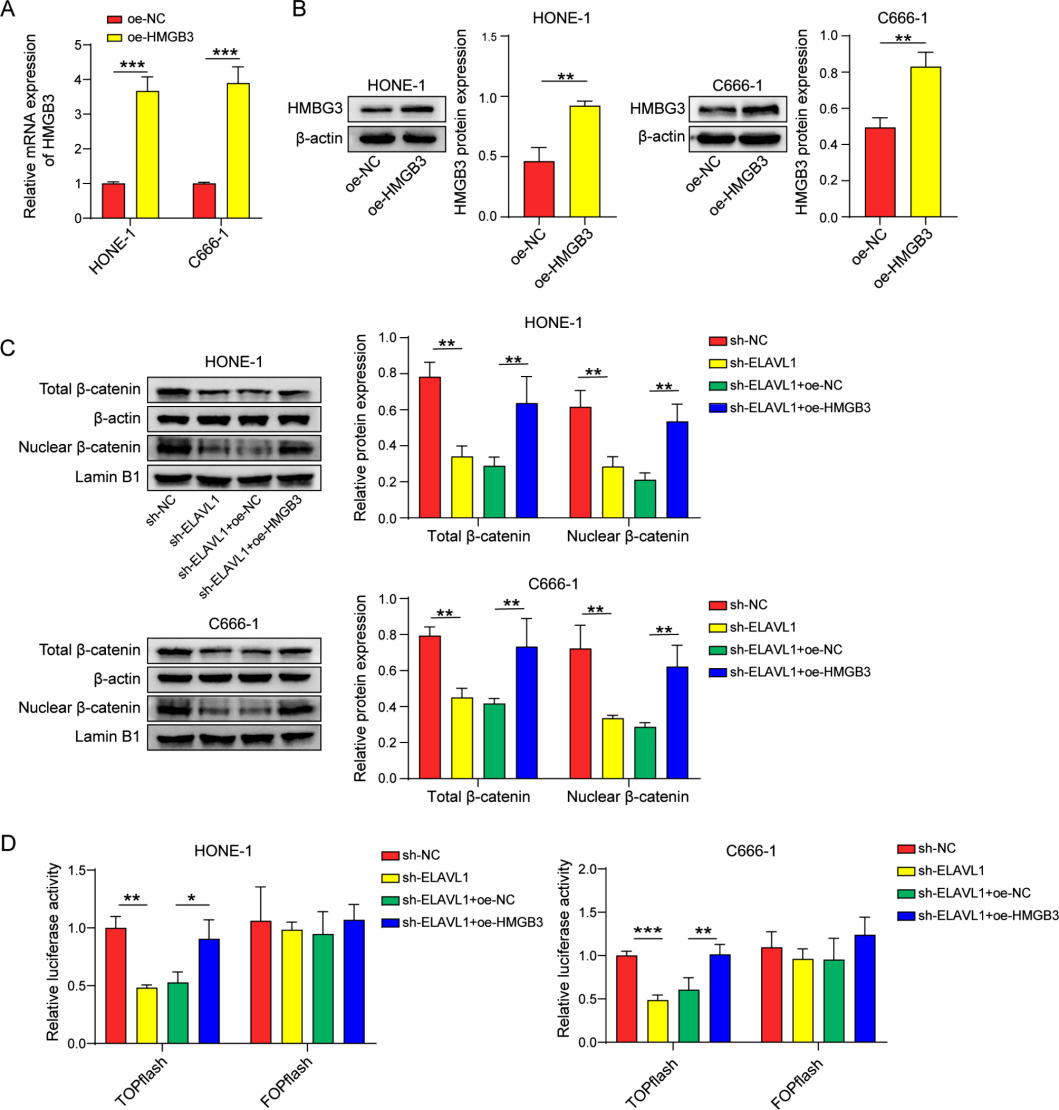
ELAVL1 activates the β-catenin pathway through HMGB3. (**A**, **B**) The mRNA and protein levels of HMGB3 were measured using qRT-PCR and western blot. (**C**) Western blot detection of total/nuclear β-catenin levels. (**D**) TOPFlash/FOPFlash reporter assay for β-catenin activity. *n* = 3. *p < 0.05; **p < 0.01; ***p < 0.001

HMGB3 was assayed to be efficiently overexpressed at both mRNA and protein levels in HONE-1 cells and C666-1 cells (Fig. 4A, B). In Fig. 4C, specific knockdown of ELAVL1 notably decreased the expression of total and nuclear β-catenin, whereas the introduction of HMGB3 partly counteracted the impact of sh-ELAVL1 on β-catenin. Furthermore, ELAVL1 knockdown alone resulted in reduced β-catenin activity, which was subsequently restored by HMGB3 overexpression (Fig. 4D).

### HMGB3 is involved in ELAVL1-mediated glycolysis in nasopharyngeal carcinoma cells

**Fig. 5:**
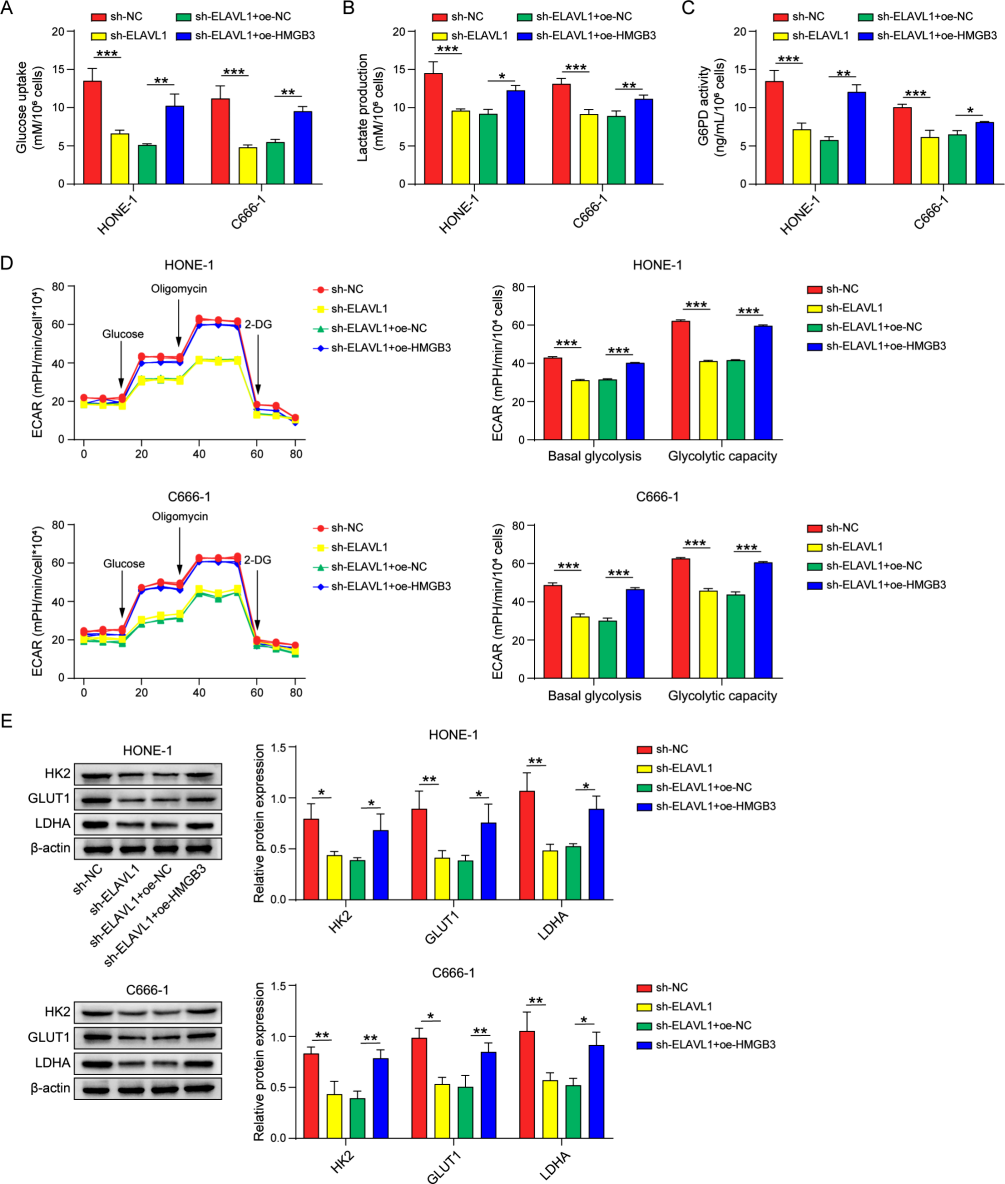
HMGB3 is involved in ELAVL1-mediated glycolysis in nasopharyngeal carcinoma cells. (**A-C**) Kits assay for glucose consumption, lactate production, G6PD activity. (**D**) ECAR analyzes cellular glycolytic capacity. (**E**) Western blot for HK2, GLUT1, LDHA protein levels. *n* = 3. *p < 0.05; **p < 0.01; ***p < 0.001

To investigate the role played by HMGB3 in ELAVL1-mediated glycolysis in nasopharyngeal carcinoma cells, we overexpressed HMGB3 in cells with knockdown of ELAVL1, and found that overexpression of HMGB3 significantly alleviated the reduced glucose consumption, decreased lactate production, and decreased G6PD activity caused by sh-ELAVL1 (Fig. 5A-C). Meanwhile, in Fig. 5D, the inhibitory effects of sh-ELAVL1 on cellular basal glycolysis and glycolytic capacity were also alleviated by overexpression of HMGB3. Moreover, the levels of glycolytic enzymes were decreased when ELAVL1 was knocked down alone and increased after concurrent overexpression of HMGB3 (Fig. 5E).

### HMGB3 promotes glycolysis in nasopharyngeal carcinoma cells through the β-catenin pathway

**Fig. 6:**
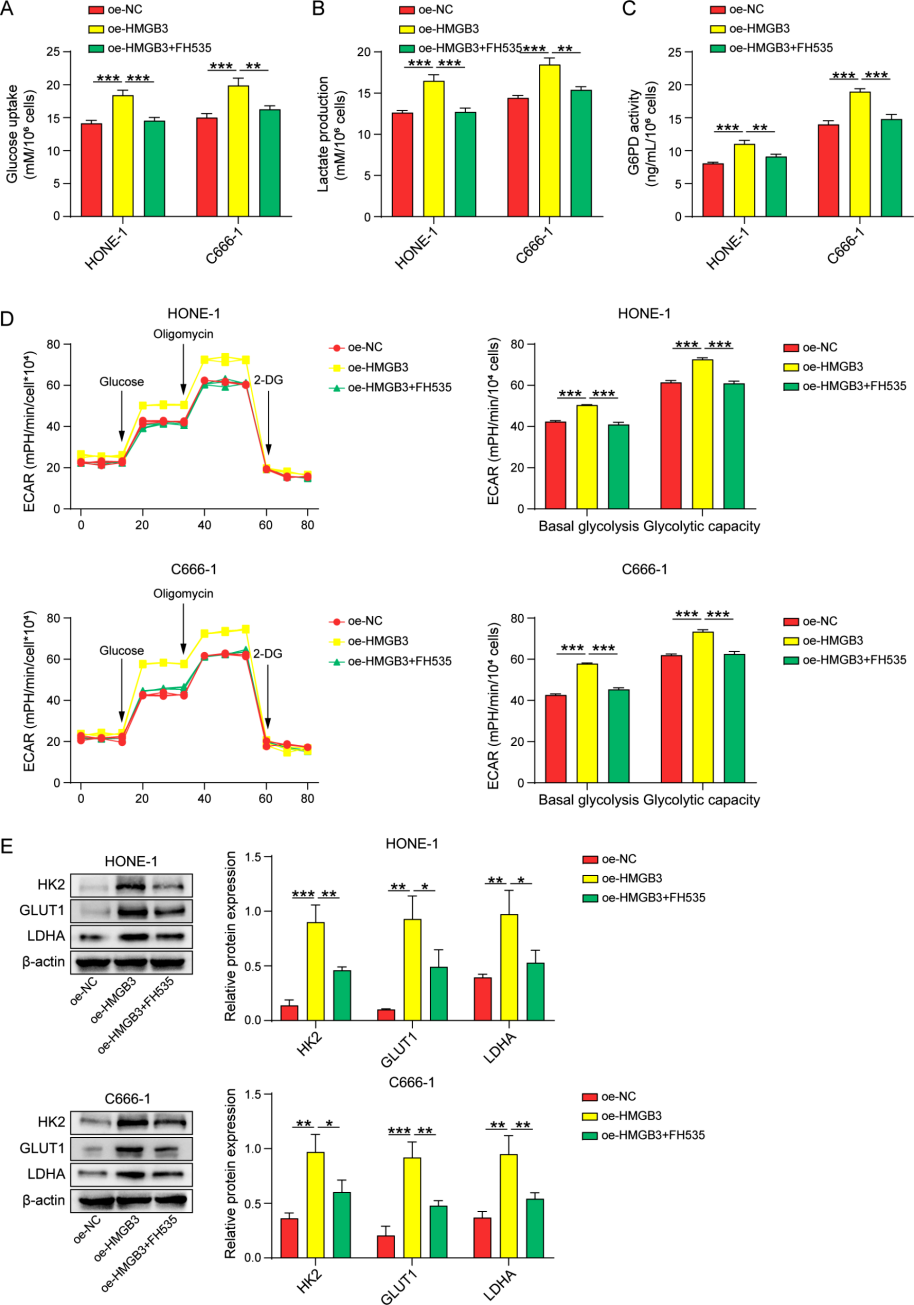
HMGB3 promotes glycolysis in nasopharyngeal carcinoma cells through the β-catenin pathway. (**A-C**) Kits assay for glucose consumption, lactate production, G6PD activity. (**D**) ECAR analyzes cellular glycolytic capacity. (**E**) Western blot for HK2, GLUT1, LDHA protein levels. *n* = 3. *p < 0.05; **p < 0.01; ***p < 0.001

Figure 6A-C demonstrates that the overexpression of HMGB3 led to a notable increase in glucose consumption, lactate production, and G6PD activity. Importantly, these effects were effectively reversed when the β-catenin signaling inhibitor FH535 was added. Moreover, FH535 exhibited a significant inhibitory effect on the promotion of basal glycolysis and glycolytic capacity induced by HMGB3 overexpression, as shown in Fig. 6D. Additionally, FH535 partially attenuated the upregulation of glycolytic marker proteins resulting from HMGB3 overexpression, as evidenced in Fig. 6E. In summary, these findings demonstrate that the overexpression of HMGB3 promotes glycolysis in cancer cells, which is attributed to the activation of the Wnt/β-catenin pathway.

### ELAVL1 promotes nasopharyngeal carcinoma progression

**Fig. 7:**
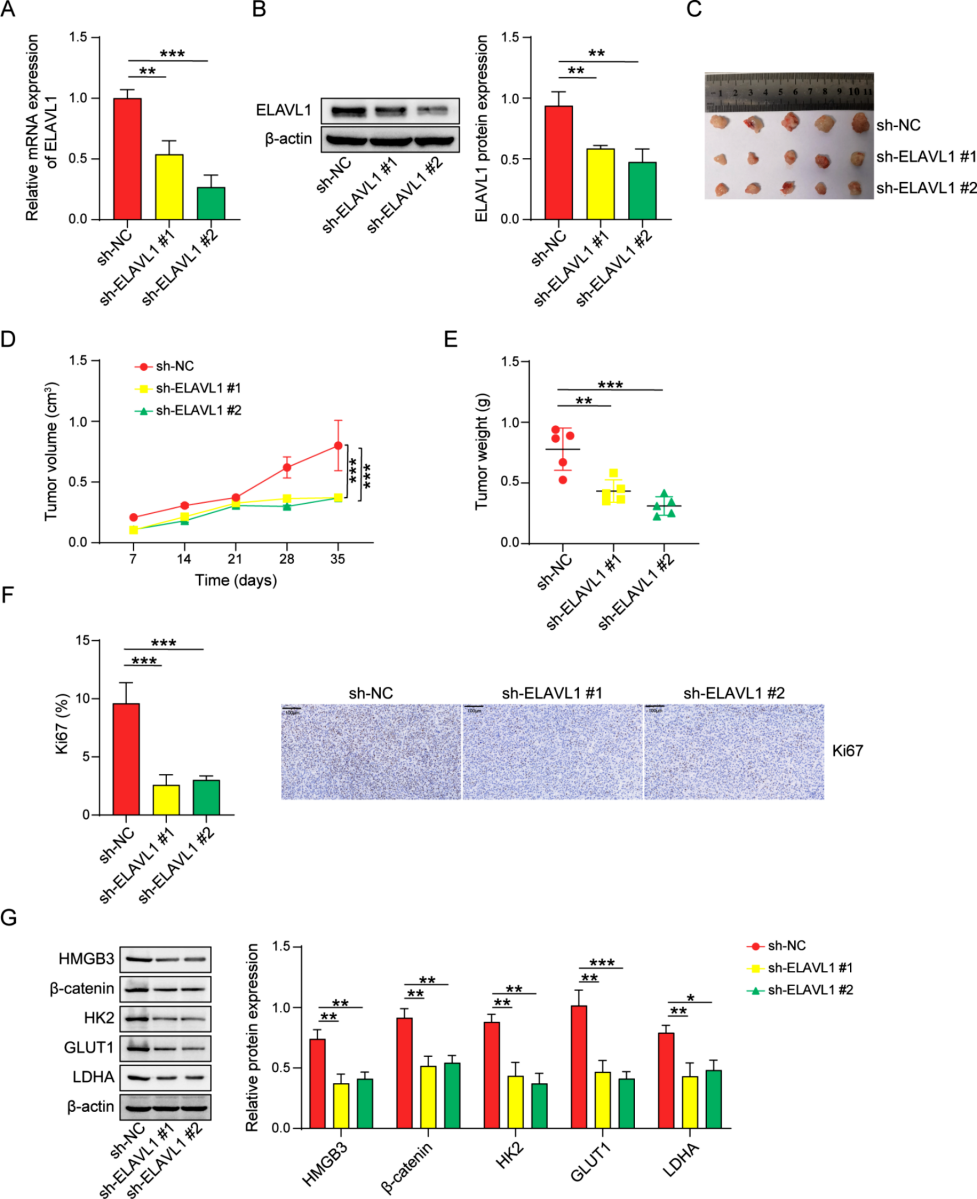
ELAVL1 promotes nasopharyngeal carcinoma progression (**A, B**) The mRNA expression and protein level of ELAVL1 in tumors of mice injected subcutaneously with HONE-1 cells were determined using qRT-PCR and western blot. *n* = 3. (**C-E**) Knockdown of ELAVL1 results in a significant decrease in tumor size, tumor growth curve and weight. *n* = 5. (**F**) Immunohistochemistry for the expression of Ki67 in nasopharyngeal carcinoma tissues of mice. (**G**) Western blot for the protein levels of HMGB3, β-catenin, HK2, GLUT1, and LDHA in tumor tissues. *n* = 3. *p < 0.05; **p < 0.01; ***p < 0.001

The functional role of ELAVL1 in tumor development was investigated by establishing stable mouse transplants with ELAVL1-silenced HONE-1 cells. The confirmation of ELAVL1 knockdown was performed using qPCR and western blot analyses. Our results demonstrated that compared to the control group, the sh-ELAVL1 group exhibited a marked decrease in ELAVL1 expression (Fig. 7A, B). Subsequently, we evaluated tumorigenic conditions in mice and observed that the downregulation of ELAVL1 remarkably hindered graft tumor growth, as indicated by reduced tumor size, tumor growth curve and weight (Fig. 7C-E). Immunohistochemical assessment of mouse tumor tissues further supported these findings, revealing a notable decrease in Ki67 expression upon ELAVL1 knockdown (Fig. 7F). Taken together, our experimental data provide compelling evidence that targeted suppression of ELAVL1 impedes tumor growth. Further, in Fig. 7G, we extracted mouse tumor tissue proteins and found that sh-ELAVL1 decreased the expression of HMGB3, β-catenin relative to sh-NC group. And sh-ELAVL1 leads to decreased expression of glycolytic markers (HK2, GLUT1, LDHA). It illustrated that ELAVL1 promoted HMGB3, β-catenin levels and facilitated glycolysis.

## Discussion

Nasopharyngeal carcinoma is a common malignant tumor in the field of otolaryngology. For countries like China, where nasopharyngeal carcinoma is prevalent, it poses a significant burden on public health and safety. Despite a decline in the age-standardized mortality rate of nasopharyngeal carcinoma in China over the past three decades, the age-standardized incidence rate has been steadily increasing. This emphasizes the importance of implementing control and prevention measures for nasopharyngeal carcinoma(Bai et al. 2022). However, the underlying mechanisms of this disease remain incompletely understood. In this study, we determined the regulatory role of ELAVL1 in the glycolytic process of nasopharyngeal carcinoma cells via the HMGB3/β-catenin axis. Our results offer the initial evidence of the interaction between ELAVL1 and HMGB3, which enhances the stability of HMGB3 mRNA. Furthermore, our findings revealed that HMGB3 has the capacity to stimulate glycolysis in nasopharyngeal carcinoma cells by activating the β-catenin pathway. These discoveries offer mechanistic insights into the involvement of ELAVL1 in the glycolysis of nasopharyngeal carcinoma and suggest potential therapeutic targets for this disease.

Initially, through data mining of clinical databases and testing in cell lines, we discovered a substantial upregulation of ELAVL1 expression in nasopharyngeal carcinoma cells compared to normal cells. This result is consistent with previous findings of elevated ELAVL1 levels in nasopharyngeal carcinoma tissues compared to non-tumor tissues (Hu et al. 2020). Furthermore, another research uncovered that individuals with elevated ELAVL1 expression had shorter overall survival and progression-free survival compared to those with low ELAVL1 expression, supporting the clinical relevance of ELAVL1 in nasopharyngeal carcinoma prognosis(Tian et al. 2023). Additionally, a substantial body of research has demonstrated a strong correlation between ELAVL1 expression and the malignant potential, tumor growth, and invasive capabilities of various types of cancer(Heinonen et al. 2011; Mitsunari et al. 2016; Richards et al. 2010; Bolognani et al. 2012; Melling et al. 2016). Our study provides additional evidence for the involvement of ELAVL1 in promoting the proliferation and progression of nasopharyngeal carcinoma.

In contrast to normal cells, tumor cells display distinct metabolic patterns, relying more on glycolysis for their energy production. This process, known as aerobic glycolysis, has emerged as a promising strategy in the development of anti-tumor drugs, as selectively inhibiting aerobic glycolysis can deprive cancer cells of their basic metabolism. Several glycolytic inhibitors are currently under investigation in clinical trials(Akins et al. 2018). However, concerns still exist regarding the potential impact on normal cell metabolism when targeting glycolysis. Therefore, the therapeutic target should demonstrate significant differences in activity or expression between cancer cells and normal cells(Abdel-Wahab et al. 2019). It has been reported that ELAVL1 can suppress the expression of HK2 and pyruvate kinase-M2 (PKM2) by specifically binding to miR-199a precursor, consequently promoting the proliferation and growth of hepatocellular carcinoma(Zhang et al. 2015). Our study found that transfection of sh-ELAVL1 into nasopharyngeal carcinoma cell lines led to a substantial decrease in glucose consumption and lactate production. Additionally, the expression of HK2, GLUT and LDHA were also markedly reduced. These findings suggest that ELAVL1 enhances glycolysis in nasopharyngeal cancer cells and may represent a potential target for targeted tumor metabolism therapy. It is noteworthy that most tumor cells also require oxidative phosphorylation of glucose for their energy supply(Ashton et al. 2018). Glucose metabolism also includes other pathways such as pentose phosphate pathway and hexosamine pathway(Hay 2016). Changes in glucose uptake may potentially affect these metabolic pathways. A recent study found that long palate, lung, and nasal epithelial cell clone 1 (LPLUNC1) act as a tumor suppressor in nasopharyngeal carcinoma, as its overexpression reduces glycolysis levels and increases expression of oxidative phosphorylation-related proteins in nasopharyngeal carcinoma cells(Oyang et al. 2023). Regarding ELAVL1, its impact on other glucose metabolism pathways in nasopharyngeal carcinoma requires further investigation.

ELAVL1, as an RNA-binding protein, plays different roles in the nucleus and cytoplasm. It binds to pre-mRNA introns and untranslated regions in the nucleus to promote mRNA maturation, while in the cytoplasm, it can stabilize mRNA, regulate its translation process, or help store it in certain granules(Assoni et al. 2022). Given its multifaceted functions, we decided to investigate the specific mechanism by which ELAVL1 regulates glycolysis. To this end, we predicted and discovered the putative binding partner of ELAVL1, HMGB3, using the ENCORI database. Subsequent experiments confirmed the interplay between ELAVL1 and HMGB3, subsequently resulting in increased expression of HMGB3. To our knowledge, this research represents the preliminary investigation illustrating the interaction between these two molecules, highlighting the roles of ELAVL1 and HMGB3 in the pathogenesis of nasopharyngeal carcinoma. HMGB3 has been reported as a positive regulator of the Wnt/β-catenin pathway in many studies(Xie et al. 2019; Zhuang et al. 2020), and the results of our own experiments support this conclusion. In addition, our experiments revealed that the suppressive impact of sh-ELAVL1 on the glycolytic ability of nasopharyngeal carcinoma cells was reversed upon subsequent overexpression of HMGB3. Moreover, the glycolytic ability promotion caused by HMGB3 overexpression was further reversed when treated with the β-catenin inhibitor FH535. These findings indicated that HMGB3 is involved in ELAVL1-mediated glycolysis in nasopharyngeal carcinoma by activating β-catenin. Interestingly, our conclusion is supported by a study conducted by Cai et al., who investigated the impact of the β-catenin antagonist Chibby on nasopharyngeal carcinoma. Their findings revealed the involvement of the Wnt/β-catenin signaling pathway in the induction of aerobic glycolysis in nasopharyngeal carcinoma cells(Cai et al. 2018). This additional perspective reinforces our own findings. Furthermore, ELAVL1 could enhance the mRNA stability of β-catenin, increase its expression at the translational level and facilitate its nuclear translocation(Huai et al. 2023). Consistently, we also found that the inhibition of ELAVL1 decreased β-catenin mNRA level (Figure S1). Therefore, ELAVL1 can activate the downstream Wnt/β-catenin signaling, either directly or indirectly, in nasopharyngeal carcinoma.

Interestingly, a study has indicated that the circular RNA circDCUN1D4 can interact with ELAVL1 in lung adenocarcinoma cells, and this interaction promotes the translocation of ELAVL1 to the cytoplasm, which stabilize TXNIP mRNA, and ultimately inhibits the glycolysis and metastasis of lung adenocarcinoma(Liang et al. 2021). This suggests that ELAVL1 has diverse functions, and its regulatory mechanisms still require further studies. Furthermore, this provides us with insights for targeted cancer therapy against ELAVL1. Due to limitations in the experimental design, we were unable to clarify whether the translocation of ELAVL1 exists in nasopharyngeal carcinoma cells. Additionally, we focused solely on evaluating the impact of ELAVL1 on the glycolysis of nasopharyngeal carcinoma cells. However, it is important to note that our study did not include additional validation of the role of glycolysis in the proliferation and metastasis of nasopharyngeal carcinoma. Future research endeavors could consider addressing these issues and further refine the research.

## Conclusions

**Fig. 8:**
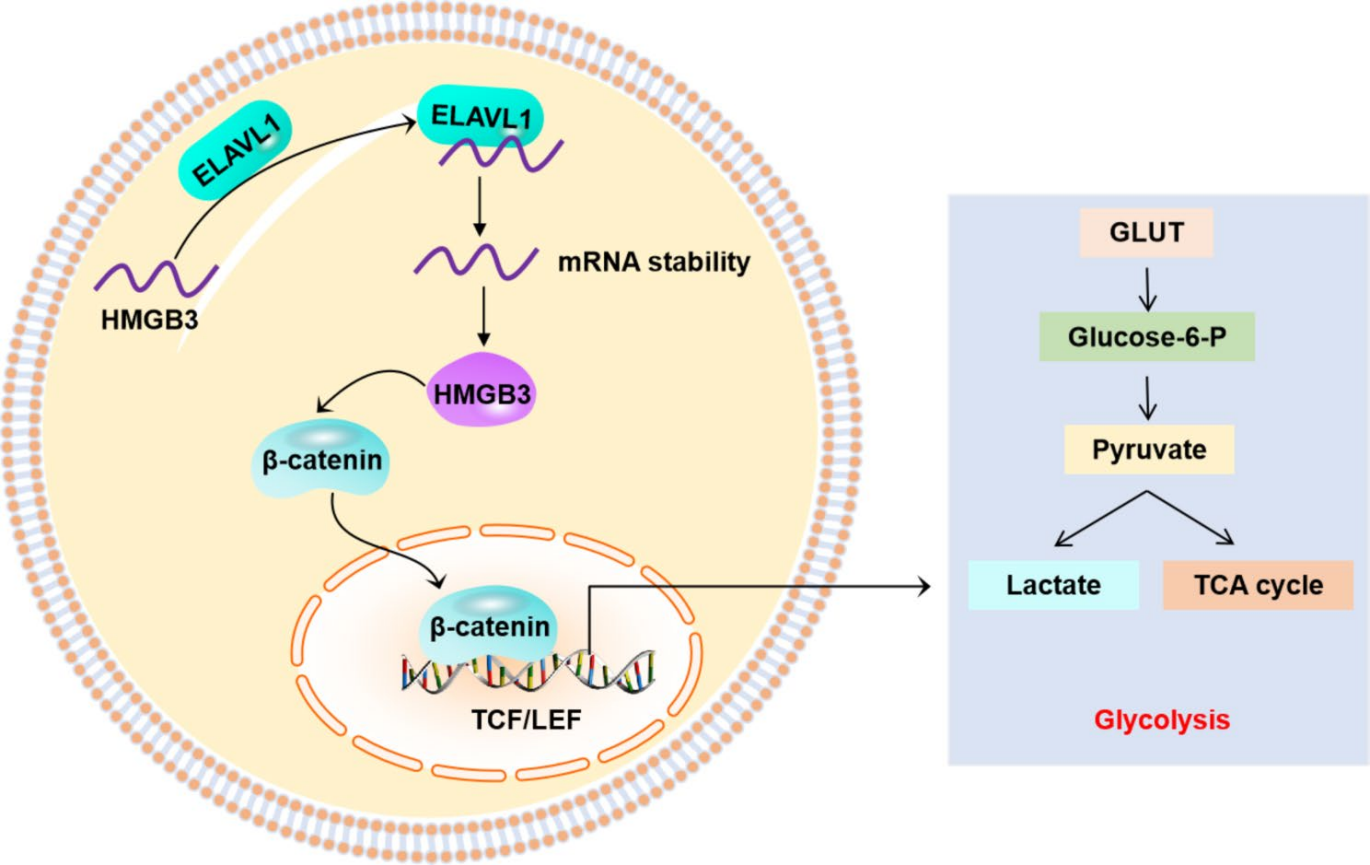
Schematic illustration showing that ELAVL1 enhances the mRNA stability of HMGB3 by interacting with HMGB3 mRNA. This interaction leads to the upregulation of HMGB3 and the activation of β-catenin pathway, thereby promoting glycolysis and proliferation in nasopharyngeal carcinoma

In conclusion, our study has confirmed the association of ELAVL1 with aerobic glycolysis in nasopharyngeal carcinoma cells. Specifically, we have identified that ELAVL1 enhances HMGB3 mRNA stability and regulates the Wnt/β-catenin signaling pathway, thereby promoting glycolysis in cancer cells (Fig. 8). Our research provides novel insights into the tumor metabolism and growth mechanisms of nasopharyngeal carcinoma, and identifies ELAVL1 as a promising potential therapeutic target for this disease.

## Electronic supplementary material

Below is the link to the electronic supplementary material.

Supplementary Material 1

## Data Availability

The datasets used or analyzed during the current study are available from the corresponding author on reasonable request.

## Abbreviations

cDNA: Complementary DNA
DMEM: Dulbecco’s Modified Eagle’s Medium
ECAR: Extracellular acidification rate
ELAVL1: Embryonic lethal-abnormal vision-like 1
ENCORI: Encyclopedia of RNA interactomes
FBS: Fetal bovine serum
GLUT1: Glucose transporter 1
HMGB3: High mobility group box 3
HK2: Hexokinase 2
mRNA: Messenger-RNA
LDHA: Lactate dehydrogenase
qRT-PCR: Quantitative real-time polymerase chain reaction
shRNA: Short hairpin RNA
