# Supplementary material for: ELAVL1 regulates glycolysis in nasopharyngeal carcinoma cells through the HMGB3/β-catenin axis
Source: Mol Med. 2024 Oct 10;30:172. doi: 10.1186/s10020-024-00941-5 (PMC11468264; doi:10.1186/s10020-024-00941-5)
Supplement: Supplementary file 1 — Supplementary Material 1 [file 10020_2024_941_MOESM1_ESM.docx]

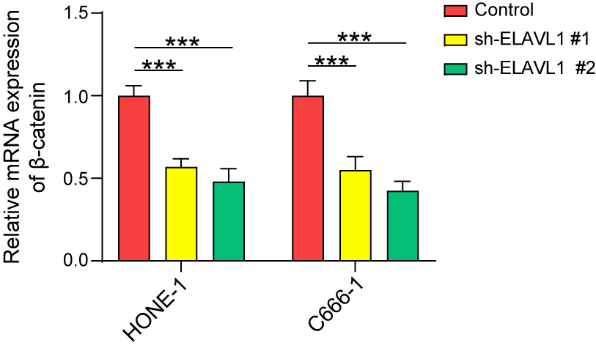


**Figure S1.** The effects of ELAVL1 knockdown on β-catenin mRNA expression. qRT-PCR assay for the mNRA expression of β-catenin.
